# Patient information, communication and competence empowerment in oncology (PIKKO) – evaluation of a supportive care intervention for overall oncological patients. Study protocol of a non-randomized controlled trial

**DOI:** 10.1186/s12874-020-01002-1

**Published:** 2020-05-15

**Authors:** Nico Schneider, Anna Bäcker, Katja Brenk-Franz, Christian Keinki, Jutta Hübner, Florian Brandt, Geraldine von der Winkel, Lutz Hager, Bernhard Strauss, Uwe Altmann

**Affiliations:** 1 Institute of Psychosocial Medicine and Psychotherapy, Jena University Hospital, Stoystrasse 3, 07740 Jena, Germany; 2 German Cancer Society, Kuno-Fischer-Strasse 8, 14057 Berlin, Germany; 3 Department of Hematology and Medical Oncology, Jena University Hospital, Am Klinikum 1, 07747 Jena, Germany; 4 IKK Südwest, Berliner Promenade 1, 66111 Saarbrücken, Germany; 5 ze:roPraxen, Bodelschwinghstrasse 10/3, 68723 Schwetzingen, Germany

**Keywords:** Cancer, Oncology, Health competence, Information, Communication, Patient navigator, Specialized oncological counseling, Oncological knowledge database, Quality of life, Self-efficacy

## Abstract

**Background:**

Cancer patients have to undergo a difficult medical therapy and are also confronted with various psychological, social and economic problems. Support is available from many providers, but patients often gain no access to it. Accordingly, there is a need for a single point of contact that can provide advice, information and assistance. In the state of Saarland, Germany, a supportive new consulting and information path (PIKKO) for all types of cancer is currently evaluated by the German Cancer Society, the Cancer Society of the Saarland, three statutory health insurances and the Jena University Hospital. PIKKO is designed to improve quality of life, self-efficacy, health literacy and patient satisfaction and to reduce psychological distress, related health care costs and the days of inability to work. This methodical work presents the process and analysis planning of this evaluation.

**Methods:**

The study population includes all cancer types, both new and existing diseases. PIKKO (with patient navigator, oncological knowledge database, specialized oncological counseling) is evaluated within a controlled, non-randomized, comparative, multicenter, longitudinal design. In addition to patient surveys, data from statutory health insurances and utilization data from the web database are collected, and interviews with patient navigators and doctors are carried out. Patients are assigned to a control (usual care) or an intervention group (u. c. + PIKKO). Primary outcome is the health related quality of life (SF-12) six months after baseline. Secondary outcomes are self-efficacy (GSE), psychological distress such as depression (PHQ-9) or anxiety (GAD-7), health literacy (HLS-EU-Q47) and patient satisfaction in health care (Qualiskope-A). Furthermore, the time course of direct costs of medical care (e.g. work disability days) and usage data of the intervention modules are analyzed. Among other statistical procedures, we use t-tests, univariate tests and growth curve models.

**Discussion:**

If PIKKO proves to be effective, recommendations can be made to health organizations, which should lead to the concept being rolled out throughout Germany and included into oncological guidelines. We expect PIKKO to be a useful addition to usual cancer care, helping to improve the quality of life of cancer patients and reduce healthcare costs.

**Trial registration:**

This study was retrospectively registered in the German Clinical Trial Register under DRKS00016703 (21.02.2019, the reason for the delay was the prioritization of the study management in the first year to establish the new approach into practice). https://www.drks.de/drks_web/navigate.do?navigationId=trial.HTML&TRIAL_ID=DRKS00016703

## Background

### Background and rationale

The treatment of cancer became more successful in the past decades. Simultaneously cancer incidence is increasing due to an aging and growing population [1]. More and more patients have to be treated and the individual survival is higher than ever before [2].

Cancer means a major change in the patient’s life and is usually associated with psychosocial distress [3]. In many cases the financial situations of cancer patients is getting worse, because patients are not or only partially able to work [4]. More than half of those affected do not feel sufficiently informed about the legal benefits to which they are entitled [4]. In addition to this psychosocial and financial burden, patients need high quality, evidence-based and helpful health information [5]. The satisfaction with respective information is related with the quality of life of patients [6]. On the other hand, regularly available health information is not suitable for laymen, especially in terms of readability and understandability [7]. This becomes even more important as patients want to actively participate in decision making concerning their therapy [8, 9], which is only possible if all relevant information on the individual medicinal situation is easy accessible and understandable. Handling complex medical information and making decisions out of them requires health competence [10] and professional advice. But there are barriers to a balanced advice from physicians, not at least because of information asymmetry and consequential communicative deficiencies between physician and patient [11–13]. In Germany, cancer patients have an average of more than 40 doctor contacts per year [14]. Many patients are treated by different specialists whereby treatment is oriented towards different national guidelines [15]. Less than 50% of patients are completely satisfied with treatment, communication, information, participation, collaboration between specialists or psychosocial aspects [16]. This calls for an adequate source of information for patients. A direct and permanent contact person for the patients and their relatives seems worthwhile.

One promising solution for a better and low threshold integration of patient needs is a patient navigation program. In 1990, Dr. Harold Freeman initiated the first patient navigation program in the USA. This early program (and other followers after the Patient Navigator and Chronic Disease Prevention Act passed by the US Congress in 2005) tried to eliminate the barriers to timely care across all segments of the healthcare continuum. A real person, a patient navigator (PN), is the most important part of these programs [17]. Usually this person is a specialized trained nurse or medical assistant.

In many cases PN are specialized to a single type of cancer e.g. breast cancer or lung cancer [18]. These concepts were accompanied by studies and showed positive effects of PN [18–20]. Fillion reports a better emotional quality of life and satisfaction in the PN exposed cohort [21]. A higher quality of life (especially physical and social functioning) in an experimental group (nurse navigation) was assessed by Lee [22]. A tendency to lower distress scores was seen regarding patients that used an oncological nurse navigator [23, 24]. A PN can decrease anxiety and increase satisfaction, e.g. after abnormal mammogram [25] or with newly diagnosed primary breast cancer [24].

To cover the complex needs of patients regarding navigation in German health care and access to evidence-based and understandable information across all cancer types a comprehensive intervention program was initiated in 2017 in Germany by the German Cancer Society, the Cancer Society of the Saarland, the Jena University Hospital and three statutory health insurance companies (IKK Südwest, Knappschaft and Techniker Krankenkasse). The program is called “Patient information, communication and competence empowerment in oncology (PIKKO)”. PIKKO supplements the usual care with a new consulting and information path.

PN have also been tested in other chronic diseases such as diabetes, AIDS, cardiovascular disease, chronic kidney disease or dementia [26]. For such cases, PIKKO could also serve as a model of feasibility in Germany.

The evaluation aims to measure the effect of PIKKO on a patient level (better quality of life, less psychological stress, better self-efficacy, better health competence, more satisfaction with the practitioners), as well as on the level of health care costs which should provide information on the implementation of the care concept and the transfer to other diseases. This paper describes the evaluation concept.

## Methods / design

### Objectives

**Fig. 1:**
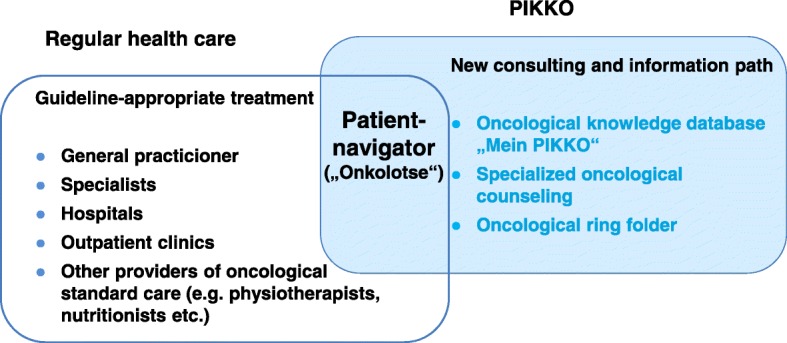
The PIKKO concept and the central patient navigator

PIKKO focuses on communicative aspects and informative needs of cancer patients and consists of four modules. The patients receive (1) the advice of a trained PN and (2) an access to a new database with evidence based information about cancer. Furthermore a fast access to a specialized oncological counseling is guaranteed (3). Finally, a ring folder is provided to patients to cope the paper flood during their illness (4). Within the project the new consulting and information path (treatment as usual plus PIKKO) is integrated to usual care (Fig. 1).

This concept is intended to become a well-established program in the German health care system for all cancer patients. But before that the PIKKO concept must be evaluated. The evaluation focuses on patients and their health-related quality of life (HRQoL) particularly the mental component. We expect that patients who underwent PIKKO report a higher HRQoL, less mental impairment such as depression and anxiety, better self-management and higher health literacy than patients of the control group. Furthermore, we expect lower direct health care costs (days of inability to work, sick-pay, expenses of health benefits).

### Trial design

The design of the evaluation study consists of four components: patient surveys, data provided by statutory health insurance companies, interviews with service providers as well as the usage data of the oncological knowledge database. The non-randomized, controlled, comparative, multicenter, longitudinal design included three groups: experimental group (treatment as usual plus PIKKO), first control group (treatment as usual; active recruited; questionnaire and health care data) and second control group (treatment as usual; random selection, only anonymous health care data). A non-randomized design with two recruitment periods, starting with the (first) control group, was chosen due to the following reasons: 1) randomization on a test person level was not possible for ethical reasons and for reasons of the statutory basis of the German health care system. 2) It is also not reasonable from a CRM (Customer Relationship Management) perspective to withhold an existing service from needy persons on the part of the statutory health insurances. 3) In a medical facility (practice, clinic), intervention and control cannot take place simultaneously to the extent required by this study (control patients could still contact PN). 4) Cluster randomization was not feasible for a project of this size and kind due to the medical infrastructure in the small state of Saarland. Accordingly, the periods were set one after the other and later the participants of the control group have the option to switch to the intervention group. Secondly, to establish the new intervention with all the descripted modules the German Cancer Society, the Cancer Society of the Saarland and the health care insurance companies needed time, nearly one year. However, no time should be lost in terms of data collection, so it was used for the patient survey of the control group. The date of recruitment determines the group assignment. The control group is included in the first project year (while the new intervention is under development) and the intervention group is recruited in the second year (when the new consulting and information path is available). A third group is a random selected sample of patients who are not part of the two other groups. Only health care data are collected for these persons. This second control group allows examining the representativeness of the sample respectively the selection process and the comparisons of treatment courses.

### Study setting

To evaluate this health care project, three participating statutory health care insurance companies (IKK Südwest, Techniker Krankenkasse, Knappschaft) work together to offer PIKKO to their insured cancer patients in the Saarland, which is the second smallest state in Germany (997,000 inhabitants). About 80 physicians (clinicians and practitioners, various specializations) in about 25 cities (different population sizes) agreed to recruit participants. The intervention is performed by specially trained PN who are located in clinical or outpatient settings.

### Eligibility criteria

#### Inclusion criteria

Patients eligible for PIKKO must comply with all of the following:

1. age ≥ 18 years and ≤ 90 years,
2. diagnosis of any cancer disease (ICD-10-diagnosis group C00-C97 or D45-D48 (initial diagnosis, relapse or transition to palliative care)),
3. current treatment by doctors from the Saarland,
4. insured with one of the three statutory health insurance companies participating in this study.

#### Exclusion criteria

Patients are not eligible in case of one of the following:

1. statutory guardianship,
2. insufficient knowledge of the German language,
3. very strong visual and hearing impairment,
4. dementia or other mental limitations.

Overall, we intend to obtain a heterogeneous sample with various types of cancer, degrees of severity, stages of disease and co-morbidities in order to reflect the reality of everyday care routine including its various aspects as well as possible.

### Recruitment

To recruit physicians to participate in PIKKO, the consortium leader (IKK Südwest) organized internal (invitations to the IKK Südwest) and participated in external (health-related congresses and similar) information events. They are addressed to all physicians of different specialization that are involved in care of cancer patients: oncologists, gynecologists, urologists, general practitioners, internists, and other. The participating hospitals, clinics, and medical practice must sign a study contract to participate in the supply concept PIKKO and to recruit patients. The enrollment is conducted by physicians or trained medical staff e.g. a nurse.

For the recruitment of PN, mailings were made to all participating hospitals, clinics and practices. Up to ten persons can be trained with a two weeks course by the German Cancer Society. Target group are people who already work in the field of cancer-related health care such as oncological nurses, social workers or other non-medical staff of an oncological outpatient clinic or oncological department of a hospital.

The patients are recruited in various ways. Firstly, they can be addressed directly while attending a medical consultation (outpatient or inpatient) as long as they show the inclusion criteria. The enrollment is carried out directly. Secondly, a landing page at the internet serves as a contact point to patients who hear about PIKKO via mass media such as newspaper articles and radio program or on various events about cancer [27]. The potential participant is using a contact form and receives a phone call by his/her health insurance company. Eligibility criteria are checked and a local enrollment is arranged. Thirdly, health insurance companies can recommend their members to attend the study. The enrollment is carried out by post. And fourthly patients can actively ask their physician, statutory health insurance, the German Cancer Society, the Cancer Society of the Saarland or the evaluator. In this case the potential participant will be led to a local enrollment person.

### Intervention

PIKKO is an additional supportive care intervention with focus on communication and information. It does not replace the usual cancer treatment. Rather, it complements the usual treatment path by integrating four new modules into care.

The central module is a PN. Within PIKKO this PN is called (in German) “Onkolotse”. This is a specially trained person with medical background but not a doctor. They attend a two week training course performed by the German Cancer Society, which was exclusively composed for PIKKO. Contents are oncological medical background incl. complementary and alternative medicine, communication, psychological oncology, nutrition and cancer, physical activity and cancer, management of side effects of cancer treatment, palliative medicine, social law, use of the oncological knowledge database, self-management and privacy. The acquired skills, the own experiences, talents and empathy qualify the PN to advise and to accompany participating patients along the complex way through their cancer treatment, especially across sectoral borders of the German healthcare system. The PN also activate the patient access to the oncological knowledge database called “my pikko” (in German “Mein PIKKO”), the second module of the intervention.

“Mein PIKKO” [27] is a web based database that provides evidence-based information across all cancer entities. The provided information is easy to understand and has been specially processed for layperson. Suitability for layperson was tested by repeated usability tests and the database was adjusted if required. On the one hand this database consists of general evidence-based information about cancer such as genesis, prevention, diagnostic, treatment incl. naturopathic ways, side effects, nutrition, physical activity, psychological support or palliative care. On the other hand there is information on socio-political aspects such as health insurance, nursing care insurance, pension insurance, accident insurance, unemployment insurance, rehabilitation or state regulations. The database is provided, hosted and consistently updated (every six month) by the German Cancer Society and includes only evidence based information and other trusted material. Other features are the search for useful addresses, prepared questions for a consultation with a physician and a dictionary. Each patient (or relatives as support) has 24/7 access to the database for quality-assured information. We expect that patient education is increasing, and therefore information gaps between physician and patient will be reduced and finally shared decision-making will be improved.

The third module of the intervention is an offer for all participating patients of a specialized oncological counseling provided by the Cancer Society of the Saarland in order to identify the psychosocial needs of cancer patients. This counseling is arranged by the PN and performed by different course leaders of the Cancer Society of the Saarland. Different offers are available: nutrition course, nutrition advice via telephone, art and creative course, music therapy, Nordic walking, QiGong, yoga, and psycho-social or psychological advices.

The forth module of the intervention was also created by the German Cancer Society. It is a ring folder, which is given to all patients at the time of their agreement. With this folder patients can collect and organize all information concerning physician or any other consulting contacts, to keep a personal medication list, to document physical complaints and to collect any discharge letter in a structured way. This folder is not only intended to improve the self-management capabilities of patients, but also to minimize informational deficits in the case of a change of care providers. In this way, patients can find other specialists and hand them over their entire treatment documentation as needed.

For any participating physician a hotline of oncologists was established. The hotline is called “onco-expert-phone” and provides the physicians fast and anytime with unbureaucratic help in case of treatment issues.

### Outcomes

#### Primary outcome

The primary outcome is the health-related quality of life (HRQoL) particularly the mental component score measured by the SF-12 questionnaire [28, 29] six months after baseline. Patients underwent intervention are supposed to improve their psychological QoL to a greater extent than patients without intervention (comparison of means of study groups). SF-12 is the valid and tested short form of the SF-36 questionnaire [30]. It is used in many cases of cancer research (e.g. [31, 32]). HRQoL is based on self-report questionnaires filled out by the patients themselves.

#### Secondary outcomes

Other measurements and supposed effects by the intervention are:

- improvement of self-efficacy measured by the General Self-Efficacy Scale, GSE [33],
- reduction of psychological distress such as depression (measured by the Patient Health Questionnaire-9, PHQ-9 [34, 35]) or anxiety (measured by the General Anxiety Disorder, GAD-7 [36, 37]),
- improvement of health literacy measured by the HLS-EU-Q47 [38, 39],
- improvement of patient satisfaction in health care measured by the Qualiskope-A [40],
- lower health care costs such as ambulatory and stationary costs, medication costs, costs of psychological therapy,
- reduction of days of inability to work (DIW).

GSE, PHQ-9, GAD-7, HLS-EU-Q47 and Qualiskope-A are well validated [29, 33, 35, 37, 39, 40]. They are part of the patient questionnaire set which will be sent to the patients. Costs and DIW are identified from secondary data analysis from the three participating statutory health insurance companies.

### Participant timeline

The enrollments of the two allocation groups take place in two consecutive enrollment periods. Every period lasts one year starting with the control group.

The patients express interest to participate in the supply concept to the enrollment person who reviews the inclusion and exclusion criteria. Furthermore, the enrollment person informs and educates the patient and hands over the oncological ring folder. After successful enrollment a standardized form is sent to the statutory health insurance company and to the evaluator. This informs the evaluator about the patient assignment and the agreement to establish contact (e.g. by telephone).

From this point in timeline there are small differences between the two groups in collecting the baseline data.

#### Control group

The evaluation team contacts the patient per telephone and informs the patient about the process of the patient survey (consent to the survey). Then the written baseline questionnaire will be sent to the patient. The patient returns the completed questionnaire postage free to the evaluator.

#### Intervention group

In the intervention group it has to be ensured that the baseline survey is carried out before the intervention starts. Due to the lack of time, the baseline questionnaire is issued directly by the PN. Before the patient and the PN start with the consultation the patient fills out the written baseline questionnaire (rated as consent to the survey). The consultation starts after the handing over of the baseline questionnaire to the PN. In addition to various consulting content and during the first consultation the PN unlocks the patient access to “Mein PIKKO” and offers the specialized oncological counseling. Later the PN returns the patient-filled questionnaire postage free to the evaluator.

In both groups the dates of the following questionnaires are computed dependent on the filling date of baseline, which is gathered directly after inclusion (T0). The patients are contacted to fill out questionnaires three (T1), six (T2), nine (T3) and twelve months (T4) after baseline, unless the patient withdraws the consent or dies, or changed from control to intervention group.

Baseline, main outcome and every follow-up survey consist of a set of questionnaires (see Table 1).

**Table 1:** Directly measured patient outcomes and time of measurement

Measure	Source	T0	T1	T2	T3	T4
Demographics	Control + intervention group	X	X			
Demographics, reduced	Control + intervention group			X	X	X
Social burden	Control + intervention group	X	X			
Social support	Control + intervention group	X	X	X	X	X
Cancer, status and treatment	Control + intervention group	X	X			
Cancer, changes	Control + intervention group		X	X	X	X
Chronic diseases	Control + intervention group	X	X			
Health-related quality of life (SF-12)	Control + intervention group	X	X	X	X	X
Self-efficacy (GSES)	Control + intervention group	X	X	X	X	X
Depression (PHQ-9)	Control + intervention group	X	X	X	X	X
Anxiety (GAD-7)	Control + intervention group	X	X	X	X	X
Patient satisfaction in health care (Qualiskope-A)	Control + intervention group	X	X	X	X	X
Health literacy (HLS-EU-Q47)	Control + intervention group	X	X	X	X	X
Nutrition	Control + intervention group	X	X	X	X	X
Alcohol use and smoking	Control + intervention group	X	X	X	X	X
Intervention modules						
Use of oncological ring folder	Control + intervention group		X	X	X	X
Utilization of PN	Intervention group		X	X	X	X
Utilization of specialized oncological counseling	Intervention group		X	X	X	X
Utilization of psychological consultation	Intervention group		X	X	X	X
Use of oncological knowledge database	Intervention group		X	X	X	X
Rating the intervention	Intervention group		X	X	X	X

#### Changing from control to intervention

During the intervention period every patient of the control group has the option to cancel the participation and to register to the intervention group. All data collected so far are assigned to the control group.

#### Additional data

The questionnaires given to the intervention patients include items to describe the use of the intervention modules.

To evaluate the impact of PIKKO on healthcare costs, all the participating health insurance companies provide pseudonymous patient data. These data are collected from three groups: control group, intervention group and a second control group of patients who meet the inclusion criteria but do not participate in PIKKO.

Furthermore, pseudonymous usage data from the oncological knowledge database are analyzed and interviews with PN and enrollment persons are carried out.

The patients agreed to the collection of these data during the inclusion. Then, these data is collected without further contact to patients and in concordance with the data protection act.

### Sample size calculation

The three participating health insurance companies have a potential of 325,000 insured persons in the Saarland and their averaged prevalence of cancer (ICD-10-diagnosis groups C00-C97 or D45-D48) is 6.532%. Thus, the study population included (rounded up) 22,000 patients.

The financing of the PIKKO supply concept was calculated for a maximum of 1.800 patients per group, 3.600 patients in total. That means that 1.800 participants of the intervention group can benefit from the PN and the other intervention modules.

To evaluate the PIKKO intervention only 676 participants (338 per group) of the patient questionnaires are required to the statistical analyses. This number of cases was calculated with the program GPower version 3.1.7 [41]. The calculation is based on an expected effect of d = 0.25 for a two-sided t-test with same sized comparative groups, a level of significance of α = 0.05 and a power of 1-β = 0.9. The t-test refers to the primary outcome. We assumed that 338 patients would drop out and added this amount to the total number of included patients. As a consequence, the recruiting aim was *N* = 1014 = 676 + 338 patients (per group 507 = 338 reaching the end point + 169 dropout). The expected dropout rate was 33.3% regarding the baseline (338 of 1014) or respectively 50% regarding the patients reached the endpoint (338 of 676).

### Data collection methods

According to the four aspects of this evaluation, four different kinds of data are obtained from four different sources.

Up to five times the patients will be asked to fill out a set of questionnaires. The length of these sets varies between 12 and 20 DINA4-pages. With the length and the allocation, the content of the set differs too (see Table 1). Questionnaire sets of the intervention group are longer than of the control group because they included questions about the frequency of use and quality of PIKKO interventions. All questionnaire sets included the six validated questionnaires SF-12 (main outcome, HRQoL, Cronbach’s α = 0.76–0.90 [29]), GSE (self-efficacy, Cronbach’s α = 0.80–0.90 [33]), PHQ-9 (depression, Cronbach’s α = 0.88 [35]), GAD-7 (anxiety, Cronbach’s α = 0.89 [37]), Qualiskope-A (patient satisfaction in health care, Cronbach’s α = 0.87–0.94 [40]) and HLS-EU-Q47 (health literacy, Cronbach’s α = 0.97 [39]). We chose the written form of patient survey because of the expected age structure of the patients. In 2013, the mean age of onset of cancer in Germany was 67.2 (female) and 68.3 (male) years [42]. In 2017, only 61% of people older than 65 used the internet privately [43], but the use of the internet is a prerequisite for online surveys. In a written survey, a much higher response rate is expected.

The second data source is data from the health insurance companies. Different types of values are summarized (see Table 2). These data are pseudonymized and cannot be matched with questionnaire data.

**Table 2:** Additional pseudonymized secondary outcomes and time of measurement

Measure	Source	Time of measurement
Economic data		
Demographics	Statutory health insurance companies	6 months after closing recruitment separated in allocation groups
Diagnoses	Statutory health insurance companies	
Ambulatory costs	Statutory health insurance companies	
Stationary costs	Statutory health insurance companies	
Costs of psychological therapy	Statutory health insurance companies	
Medication costs	Statutory health insurance companies	
Days of inability to work	Statutory health insurance companies	
Oncological knowledge database		
Usage data: number of visits, visited subpages, printed content, online time	German Cancer Society	6 months after closing recruitment of the intervention group

Log files from “Mein PIKKO” are the third data type. This anonymous data will be provided from the German Cancer Society and based on the usage of the oncological knowledge database (see Table 2).

The last data types are telephone interviews carried out with PN and study physicians. These interviews focus on quality and implementation aspects of the PIKKO intervention.

#### Participant withdrawal and other drop-outs

Patients may withdraw from the participation for any reason at any time. The patients are not obliged to justify their withdrawal. However, they will be asked about it. Possible reasons may be deterioration of health or consideration of the questionnaires as an additional burden. If the patient does not return the written questionnaire set, we contact the appropriate enrollment person to identify the reason.

### Data management

#### Data organization

Contact and process information are saved in a database which is programmed with Microsoft Access especially for this evaluation (names; address; telephone number; allocation; contact events, outgoing or incoming of questionnaires; date of enrollment; date of dispatch; date of fill out; enrollment person; PN; withdrawal; data entry). The database keeps track the patients’ timeline and allows intervening if the time interval between the patient questionnaires becomes too large (algorithm and alerts). In addition, the database enables the cooperation partners to be kept up to date in terms of recruitment and study process (reports and summaries). Access is multiple times secured for privacy reasons.

#### Data entry and quality

The filled-out questionnaires are scanned with a standard hardware (Fujitsu fi-7140) and analyzed with FORMS Desktop (Lexmark Enterprise Software) which is software to automatic data entry. This procedure allows a fast and accurate data entry. All marks in checkboxes are transferred with a security of 100%. Numeric and alphabetical characters are verified by the study assistant. During the period of data entry, permanent controls and plausibility checks are performed by the data manager.

#### Data security

All electronic data is located in a secure folder on a secure intranet. Only members of the evaluation team get access. The organization database is restricted by passwords. Electronic data is protected against loss by a security procedure of the Jena University Hospital.

According to the German laws regarding clinical studies the paper questionnaires will be kept for 10 years in a safe archive of the Jena University Hospital.

### Statistical methods

Characteristics of control and intervention groups (e.g. age, sex, education, family situation, partner situation) will be analyzed with descriptive statistics. Since no randomization is made for ethical and research practical reasons, univariate tests are used to determine which of the above mentioned characteristics differ between the two groups. Ideally there are no significant differences between the two groups. In the case of significant differences we will compute propensity scores using the corresponding pre-treatment variables. Then, we will apply in the subsequent analyses propensity scores as stabilized regression weights to reduce bias caused by different group characteristics.

The main hypothesis of an intervention effect on psychological and physical health, self-efficacy, health care costs etc. will be proofed by the comparison of averages of intervention and control group (resp. t-tests). To estimate the corresponding regression adjusted group means we will use growth curve models. Furthermore, we will conduct subgroup analyses to identify conditional treatment effects.

For dropout analysis we will conduct a logistic regression with dropout as criterion and group assignment and baseline variables as predictors applying the regression weight mentioned above. For the time-to-event analysis we will conduct a Cox regression.

The level of significance will be α = 0.05. To validate our analyses, we will conduct the analyses with imputed missing data and without imputation.

### Imputation procedure for missing data

We expect the occurrence of missing values due to dropout or not completely filled in questionnaires. For imputation of missing data we will use a nonparametric approach by random forest imputation respectively the R package missForest [44, 45]. The method showed better results in comparison to the Hot Deck technique, mean imputation, or multivariate imputation [46, 47]. According to MacNeil et al. [48] and Altmann et al. [49] the cost data are transformed logarithmically before imputation because they are not normally distributed and zero-inflated. After imputation, the cost data will be retransformed.

### Monitoring

The numbers of enrolled patients and other organizational data are monitored and sent weekly to the consortium leader (IKK Südwest). The study progress is reported quarterly according to an approved milestone plan. All events, progress data, milestones and failures are reported annually to the sponsor. The PN are supervised quarterly by the Cancer Society of the Saarland.

### Adverse events

PIKKO is a supportive care element without invasive impact on the patients.

## Discussion

So far, there is no other project in Germany that offers cancer patients such a wide range of possibilities as PIKKO. There is also no known international study that combines PN, technical applications and course offerings. There are many technical applications, from websites [50] to apps [51, 52]. Patient navigators are also being tested [53, 54]. There are also numerous cancer advice centers [55, 56] and courses for cancer patients [57]. To bundle all these services and offer them together is an innovation of this study. Finally, the strengths and limitations of such an extensive project should be noted.

### Strengths

This new supportive care concept is intended to establish an offer for all cancer patients. Low barriers for the procurement of information such as the website, which offers both specific and general information, are just as much a part of the concept as the broad further training of PN, who can thus advise all cancer patients.

The study is designed to evaluate the effect of PIKKO interventions under naturalistic conditions. The according heterogeneity of the sample (age range, all cancer types, all sexes) tries to reflect the reality of oncological care. Due to the heterogeneity of the sample, subgroup analyses are planned to show which module of PIKKO works best for whom.

Furthermore, the longitudinal design with five measurement points allows the investigation of long-term effects.

Last, the telephone interviews with participating PN and physicians can show experiences in the implementation that help to make PIKKO a common part of cancer care.

### Limitations

In general, less burdened patients, patients with a stronger self-motivation, patients with higher self-efficacy or patients with more social support could preferably participate, leading to a selection bias. Related with this bias can be an overestimation of treatment effects when the treatment is especially effective for less burdened patients. Also an underestimation of the mortality or patient’s withdrawal is possible. To examine the selection bias, we included a second control group which is a random sample of eligible patients based on health insurance company data. We expect that the selection bias is small so that the generalizability of study results should be high.

The voluntary and selective participation of hospitals (respectively individual departments), clinics and practices could lead to a selective clientele of patient e.g. disproportionately many breast cancer patients. However, this could also make the study more comparable to the many studies that have been pre-determined for certain groups, e.g. specialized in breast cancer patients [18].

Due to ethical and other reasons (described in the section “Trial design”) the design is not randomized. To compensate for this limitation, we will use propensity scores for analyses [58, 59].

Since we will collect questionnaires from seriously ill and elderly patients, we might expect incomplete questionnaires. Missing items and missing complete questionnaires should be compensated by imputation of missing data [44, 45].

Each participant agrees with the use of his data from the statutory health insurance companies. These data are collected from all included patients without their direct participation. However, to collect questionnaire data, we rely on the direct support of the patients. The number of data sets can therefore vary between data from the questionnaires and from the statutory health insurance companies.

### Trial status

The study protocol was approved by the ethics committee of medical association of the Saarland at 2nd November 2017. The first patient of the control group was included on 23th November 2017. The control group was recruited till 31st October 2018. Since then till 31st March 2020 the intervention group was recruited. An extension of the recruitment period of the intervention group (from one year to one and a half year) was necessary due to the entry of a further statutory health insurance (AOK Rheinland-Pfalz/Saarland) in the summer of 2019.

## Data Availability

Not applicable.

## Abbreviations

DIW: Days of inability to work
GAD-7: General Anxiety Disorder
GSES: General Self-Efficacy Scale
HLS-EU-Q47: European Health Literacy Survey Questionnaire
HRQoL: Health-related quality of life
PHQ-9: Patient Health Questionnaire-9
PIKKO: Patient information, communication and competence empowerment in oncology
PN: Patient navigator(s)
SF-12: Short form 12 health survey
